# A step towards international prospective trials in carbon ion radiotherapy: investigation of factors influencing dose distribution in the facilities in operation based on a case of skull base chordoma

**DOI:** 10.1186/s13014-019-1224-1

**Published:** 2019-02-01

**Authors:** G. Vogin, A. Wambersie, M. Koto, T. Ohno, M. Uhl, P. Fossati, J. Balosso, on behalf of ULICE WP2 working group, Richard Pötter, Michael Beuve, Stephanie E. Combs, Giulio Magrin, Ramona Mayer, Ulrike Mock, David Sarrut, Thomas Schreiner

**Affiliations:** 1 Department of Radiation Oncology, Institut de Cancérologie de Lorraine, 6 avenue de bourgogne - CS 30519, 54519 Nancy, Vandoeuvre-les-Nancy Cedex France; 2 UMR 7365 CNRS-UL, IMoPA, Nancy, Vandoeuvre-les-Nancy Cedex France; 3 Institut de Recherche Expérimentale et Clinique (IREC), Molecular Imaging, Radiotherapy and Oncology (MIRO), University Clinics St Luc, Brussels, Belgium; 4 Université catholique de Louvain (UCL), Louvain-la-Neuve, Belgium; 5 Hospital of the National Institute of Radiological Sciences, National Institutes for Quantum and Radiological Sciences and Technology, Chiba, Japan; 6 Gunma University Heavy Ion Medical Center, Gunma University, Maebashi, Gunma Japan; 7 Universitätsklinik Heidelberg, Abteilung für Radioonkologie und Strahlentherapie, Heidelberg, Germany; 8 EBG GmbH MedAustron, Wiener Neustadt, Austria; 9 Fondazione CNAO (Centro Nazionale di Adroterapia Oncologica), Pavia, Italy; 10 Service de Cancérologie-Radiothérapie, Hôpital A.Michallon, CHU de Grenoble, Grenoble, France; 11 Université Grenoble Alpes, Grenoble, France; 12 Département de radiothérapie, Centre François Baclesse, Caen, France

**Keywords:** Carbon ion radiotherapy, Equieffective dose, Clinical trials methodology, Chordoma

## Abstract

**Background:**

Carbon ion radiotherapy (CIRT) has been delivered to more than 20,000 patients worldwide. International trials have been recommended in order to emphasize the actual benefits. The ULICE program (Union of Light Ion Centers in Europe) addressed the need for harmonization of CIRT practices. A comparative knowledge of the sources and magnitudes of uncertainties altering dose distribution and clinical effects during the whole CIRT procedure is required in that aim.

**Methods:**

As part of ULICE WP2 task group, we sent a centrally reviewed questionnaire exploring candidate sources of uncertainties in dose deposition to the ten CIRT facilities in operation by February 2017. We aimed to explore native beam characterization, immobilization, anatomic data acquisition, target volumes and organs at risks delineation, treatment planning, dose delivery, quality assurance prior and during treatment. The responders had to consider the clinical case of a clival chordoma eligible for postoperative CIRT according to their clinical practice. With the results, our task group discussed ways to harmonize CIRT practices.

**Results:**

We received 5 surveys from facilities that have treated 77% of the patients worldwide per November 2017. We pointed out the singularity of the facilities and beam delivery systems, a divergent definition of target volumes, the multiplicity of TPS and equieffective dose calculation approximations.

**Conclusion:**

Multiple uncertainties affect equieffective dose definition, deposition and calculation in CIRT. Although it is not possible to harmonize all the steps of the CIRT planning between the centers, our working group proposed counter-measures addressing the improvable limitations.

## Highlights

- CIRT should be reported similarly to initiate international trials
- Different uncertainties affect dose-effect relationship throughout CIRT planning
- Equieffective dose and target volume definition and application require particular attention
- We propose some suggestions to harmonize the practices

## Background

Carbon ions are densely ionizing particles with a higher relative biological effectiveness (RBE) and physical selectivity compared to photons [1, 2].

Carbon-ion radiation therapy (CIRT) is available in 10 centers worldwide [3]. While Japanese centers have studied a broad spectrum of indications with dose escalation trials, hypofractionation and passive beam shaping (PS), the German facilities have treated smaller but homogeneous cohorts, developed fully active beam shaping (ABS), and a specific mapping of RBE-weighted dose. Per end of 2017, more than 20,000 patients have been treated worldwide with CIRT, mainly in phase I-II trials [4–7].

Due to the technical and financial investments required to expand CIRT, the decisions-makers are requesting phase III studies. These trials are particularly complex to lead in CIRT due to the rarity of indications and available resources, but also due to the different practices and environments of the dedicated centers [8, 9].

To face these challenges, it is critical that the CIRT treatments be reported similarly. Experts from IAEA and the International Commission on Radiation Units and measurements (ICRU) met in 2004 and 2006 to address these issues and published dedicated proceedings [10]. However, there is still no official recommendation.

### The ULICE project

In this framework, ULICE was a FP7 project (part of ENLIGHT) initiated in 2009 to address specific issues such as particle radiobiology, intra-fractional moving targets, adaptive treatment planning, database organization and gantry engineering [11]. In particular, ULICE-WP2 aimed to adapt joint concepts for dose volume and outcome assessment, standard operating procedures for clinical trial design and a clinical research infrastructure.

The objective of our study was to describe and compare each stage of the treatment process in the existing CIRT centers worldwide illustrated in a case of skull base chordoma (SBC).

SBC is a rare and slow growing malignancy arising from the remnants of the notochord, optimally managed with surgery and postoperative radiation [12]. SBC is one of the most consolidated indications of CIRT [13–17].

## Methods

ULICE-WP2 included the following members: radiation oncologists involved in CIRT either in treating (*n* = 3) or in planned facilities (*n* = 6) or in ICRU (*n* = 1), physicists (*n* = 6), computer scientist expert in imaging (*n* = 1) or machine learning (n = 1), radiation biologists (*n* = 2) and quality manager (n = 1). Based on ten working parties, we produced a set of consensual reports released to the European Commission after a formal approval procedure involving the Steering Committee, the Technical Project Board and an External Scientific Advisory Board [18–23].

We drafted a survey addressing the potential sources of uncertainties altering clinical effects as far we may hypothesize**,** based on ICRU and our own reports [4, 10, 19–28] (Table 1). The questionnaire was centrally reviewed within the group and sent to the expert radiation oncologist(s) involved in SBC management in the 10 CIRT facilities in operation by February 2017.

**Table 1:** Outline of the survey

∙ Institution/name	
∙ Equipment	
o type of accelerator,	
o energy,	
o beam delivery system,	
∙ Treatment setup	
o patient position: *lying, sitting*,	
o immobilization, fixation device(s) used	
o methods used to ensure positioning reproducibility	
o methods used for recording patient positioning: *XR, scan*	
o image fusion (if used): technique, recording	
∙ Delineation procedure	
o procedure for CTV(s) delineation	
o safety margins added around the CTV to define the PTV and to ensure its proper coverage	
o do you proceed separately for each beam to add the above-mentioned margins?	
o procedure for dose prescription/specification to the CTV-PTV	
o procedure for dose limitation/specification to OARs	
∙ Beam delivery	
o one single fixed beam	
o several fixed beams	
o scanning beam	
∙ Dosimetry	
o biological plan optimization	
o beam dosimetry: calibration, homogeneity	
∙ Procedure for control and validation of the treatment plan before application to the first session	
∙ What kind of record are stored at the end of the treatment?	

**Fig. 1:**
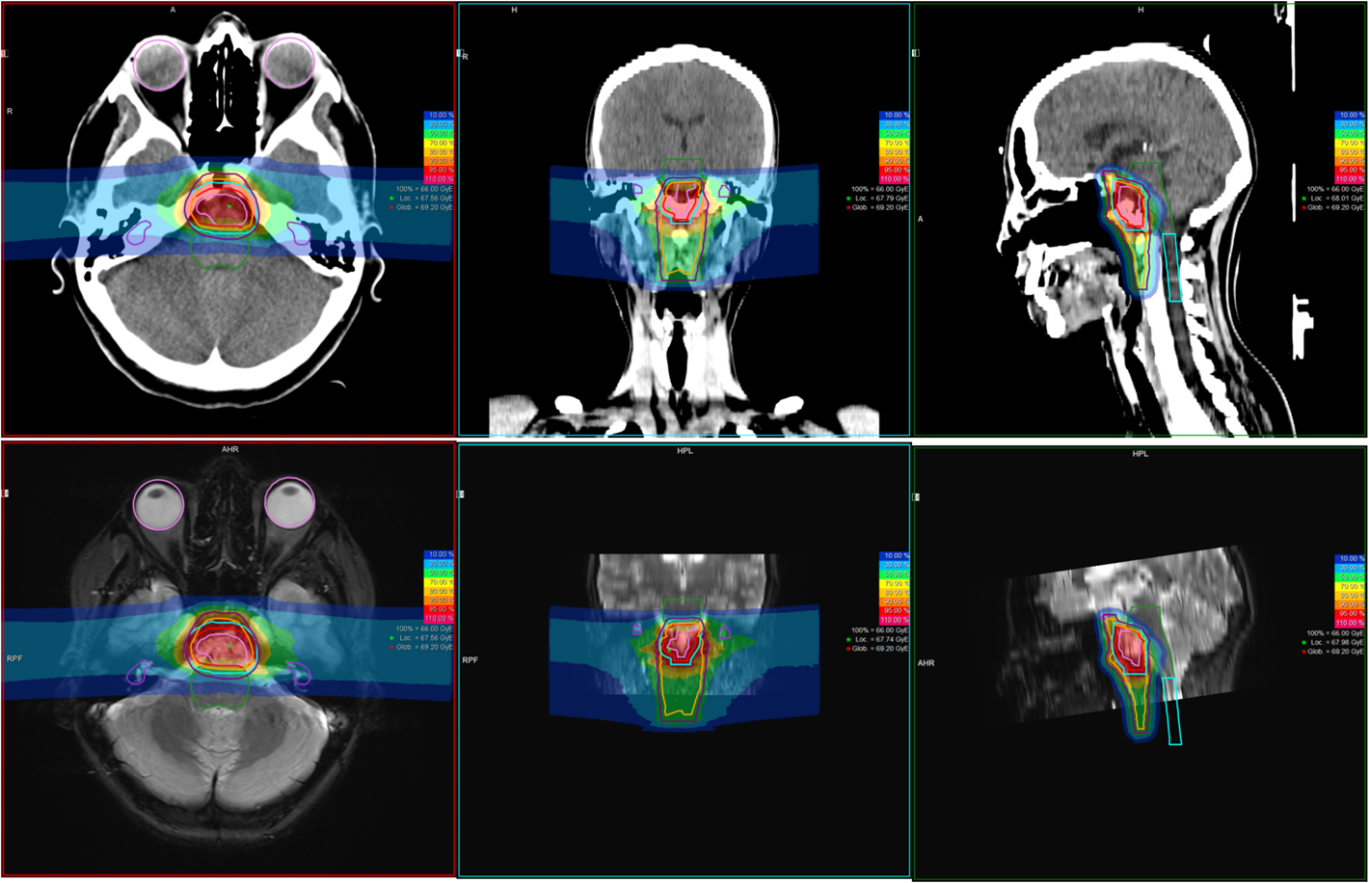
Clinical case of a clival chordoma treated with CIRT; Primary + Boost plan 66Gy (EQD) in 22 Fx. Top panel: CT scan; Bottom panel: T2w MRI (courtesy Dr. Uhl, HIT)

The responders had to consider the following clinical case: a 50 y.o. man with a clival chordoma previously operated with a macroscopic residual tumor (R2) and eligible to one the following state-of-art CIRT schedules:

A) 45 Gy EQD in 3 Gy EQD/d, 5 days a week, 15 fractions on the initial PTV (PTV1) including the GTV and suspected subclinical disease – followed by a boost to a cumulative dose of 63–66 Gy EQD in 3 Gy EQD/d, 5 days a week, by 6–7 additional fractions, on a restricted PTV2 including the GTV visible on the MRI [29] (Fig. 1).

B) 34.2–39.6 Gy EQD in 3.8–4.4 Gy EQD/d, 4 days a week, 9 fractions on the PTV1 – followed by a boost to a cumulative dose of 60.8–70.4 Gy EQD in 3.8–4.4 Gy EQD/d, 4 days a week, by 7 additional fractions on the PTV2 [30].

## Results

We received 5 surveys from seven facilities representing 77% of the patients treated worldwide per November 2017 (16,704 pts. out of 21,675, Table 2A). HIT and MIT use exactly the same equipment and procedures and we distinguished HIMAC “old” and “new” facilities. We analyzed the various hypothesized sources of uncertainties.

**Table 2:** Main results in the participating centers

Country	Institution	A Pt Statistics		B Equipment								C Positioning and immobilization devices		D Definition of the target volumes					E Prescription schedule(s) and dose constrains to OARs				F Treatment planning system and dose calculation		G Beam delivery system and positioning control		
		Opening date	Total patients treated (per nov 2017)	Ion source	Injector	Type of accelerator, diameter	Manufacturer	Max C ions energy (MeV/u)	Max beam Intensity (C/spill) and Repetition rate (Hz)	Max field size	Treatment room(s)	Patient position	Immobilization device	Image fusion?	GTV definition	Procedure for CTV(s) delineation	CTV-PTV margin	Portal-specific PTV?	Treatment schedule	Dose unit reported in the original protocol	Dose prescription for PTV	Dose constraints for OAR	TPS	Biological modeling	Beam delivery System	Beam energy	Methods to ensure positioning reproducibility
Japan	Chiba (HIMAC) pilot facility	1994	10,692	10 GHz Kei2 ECR, 18 GHz ECR & PIG	RFQ + Alvarez linac	Dual synchrotron, 42 m	Research machine	400	1.2 × 10^9 0.3 Hz	22 cm	3 (1H, 1 V, 1H&V)	Lying (Supine position)	Customized cradles (Moldcare®), thermoplastic mask, Vacuum bags for the body	2D to 2D	T1 post gadolinium and T2 (fat sat or Flair fat sat)	CTV1 = CTV2: minimum margin of 5 mm around the pre-op GTV. Only one CTV and one dose level applied	2–3 mm	yes	B	GyE	16 fractions, 4 days a week over 4 weeks. The target reference point dose is defined as the isocenter, and the PTV is encompassed by the minimum 90% dose line of the reference point dose.	Brain stem: Dmax ≤30 Gy EQD; Optic pathway: Dmax ≤40 Gy EQD; Temporal lobe: V50 ≤ 5 cc	Xio-N (ELEKTA and Mitsubishi Electric, Tokyo, Japan) + K2DOSE;	Biological adjustment with HSG cell line Modified MKM	passive conventional and spiral beam wobbling	290–400	Orthogonal X-ray images
	Chiba (HIMAC) new facility	2011					Toshiba	430	6 × 10^9 0.3 Hz	22 cm (gantry: 20 cm)	+ 3 (2H&V, 1 gantry)														active Pencil-beam 3D scanning	290–430	
	Gunma (GHMC)	2010	2231	10 GHz KeiGM ECR	RFQ + APF linac	Synchrotron, 20 m	Mitsubishi Electric	400	1.2 × 10^9 0.5 Hz	15 cm	3 (1H, 1H&V, 1 V)	Lying (supine, prone, or lateral) position with rolling depending on tumor location and beam direction		2D to 2D		CTV1: pre-op GTV + a margin of 3-5 mm including suspected subclinical disease CTV2: same as GTV visible on MRI	2 mm	no							Passive (Single or Spiral Wobbling), Layer-Stacking technique available	290–400	
Germany	Darmstadt (GSI)	1998–2009	440	14.5 GHz CAPRICE ECR	RFQ+ IH-DTL + Alvarez (UNILAC)	Synchrotron, 20 m	Research machine	430	1 × 10^8 0.1–0.5 Hz	20 cm	1H	/	/	/		/	/	/	/	CGE	/	/	/	/	active raster scanning, intensity modulated	/	/
	Heidelberg (HIT)	2009	2430	14.5 GHz Supernanogan ECR ×2	RFQ+ IH-DTL linac	Synchrotron, 20 m	GSI and Siemens	430	1 × 10^9 0.3 Hz	20 cm	3 (2H, 1 gantry)	Lying (Supine position)	Thermoplastic mask and individual mouthpiece	2D to 2D and 2D to 3D		CTV1 (primary plan): pre-op GTV + whole clivus + prevertebral muscles down to C2 CTV2 (Boost plan): postop GTV + 2 mm	3 mm	no	A	Gy_E	22 fractions, 5 (MIT) or 6 (HIT) days a week over 3.5–4.5 weeks; coverage of the PTV with the 95%-isodose line of the prescribed dose. Dose specification is based on equieffective dose	Optic pathways: Dmax ≤50 Gy EQD; Brainstem surface*: Dmax ≤54 Gy EQD *1% of the volume	Syngo inverse RT Planning (Siemens, Erlangen, Germany)	LEM		50–430	Orthogonal x-rays or cone-beam-CTs
	Marburg (MIT)	2015	95	14.5 GHz Supernanogan ECR ×2	RFQ+ IH-DTL linac	Synchrotron, 20 m	Siemens	430	1 × 10^9 0.3 Hz	20 cm	4 (3H, 1 45 deg)																
Italy	Pavia (CNAO)	2012	816	14.5 GHz Supernanogan ECR ×2	RFQ+ IH-DTL linac	Synchrotron, 24.5 m	Prototype	480	4 × 10^8 0.3 Hz	20 cm	4 (3H, 1 V)	Lying (Supine position), if needed with head rotation	Customized rigid non-perforated thermoplastic-masks, mouth-bites and head-rests and/or moldable body-pillows	2D-3D automatic fusion		CTV1 (low dose): pre op GTV plus 5–10 mm margins excluding optic chiasm and brainstem, but including surgical routes and prevertebral muscles. Caudal level determined on a case by case basis. CTV2 (high dose): 5 mm expansion from post op GTV, excluding brainstem and optic chiasm, including whole clivus and eventually cavernous sinus	2 mm	no**?**	A or B	Gy [RBE]	Treatment planning aims to the coverage of the PTV with the 95%-isodose line of the prescribed dose. Dose specification is based on equieffective dose	Schedule A: optic pathways: Dmax ≤53 Gy EQD; brainstem: Dmax ≤55 Gy EQD; one cochlea: Dmax ≤45 Gy EQD. Schedule B: optic pathways: Dmax ≤40 Gy EQD, D20% ≤ 28 Gy EQD; brainstem: Dmax ≤35 Gy EQD; one cochlea: Dmax ≤45 Gy EQD	Syngo inverse RT Planning (Siemens, Erlangen, Germany) + RayCarbonPlanning module (RaySearch Laboratories AB, Sweden)	LEM	active raster scanning, intensity modulated	115–400	Optoelectronic pre-alignment with infrared reflecting beads and cameras, daily orthogonal X-ray and 2D-3D fusion; in-room optical tracking system (OTS) and patient verification system (PVS)
**TOTAL**			16,704																								

Column A: patient statistics (source PTCOG website); Column B: Equipment; Column C: Positioning and immobilization devices; Column D: Definition of the target volumes; Column E: Prescription schedule(s) and dose constrains to OAR; Column F: TPS and dose calculation; Column G: Beam delivery system and positioning control

### Native beam characterization

According to Table 2B, different equipment has been set up in the successive facilities since 1994. In Japan, the two synchrotrons of the pilot Heavy Ion Medical Accelerator in Chiba (HIMAC) produce ion beams from ^4^He to ^54^Xe up to a maximum energy of 800 MeV/u. PS was first implemented with a beam-wobbling and ridge filter delivery method. The same type of facility has been reproduced in Hyogo, Gunma and Tosu, making the PS delivery method the most widely used in the world so far (Table 2). At the same time, GSI reproduced and improved the ABS system originally outlined in the pioneer facility in Berkeley - standardizing the raster scanning delivery system in European then Chinese facilities. More recently the new HIMAC facility implemented a high dose rate ABS system incorporating superconducting technology, which offers fast 3D repainting as well as a reduced gantry.

### Anatomic data acquisition

Patient setup may interfere on intra/inter fraction tumor motion and the related CTV-PTV margin to apply (Table 2C).

#### Patient position

Patients are usually treated lying in supine position - with rolling up to 20° when gantries are not available. They can also be irradiated in prone or lateral position.

#### Immobilization device

In the Japanese centers, the patients are immobilized with a 3 mm-thick non-perforated thermoplastic mask over a MOLDCARE™ pillow. The mask is wrapped around the patient-pillow-cradle block and fixed with straps. Vacuum bags are used to maintain the rest of the body.

The German centers use preferentially 3-point soft continuous thermoplastic masks with individual dental splints and a set of standardized head supports.

CNAO resort to customized rigid non-perforated thermoplastic-masks, mouth bites and head-rests or moldable body pillows.

#### Simulation CT scan

All facilities acquire anatomic data with a CT scan with (Japan, Germany) or without (CNAO) intravenous contrast. The CT slice thickness is less than 3 mm. Hounsfield units from the simulation CT scan as well as from the mask need to be converted in relative stopping powers to accurately define water equivalent tissue thickness regarding the range of the particles.

### Delineation

#### GTV definition - imaging - fusion

All the centers perform image fusion with MRI sequences and non-deformable algorithms based on anatomical landmarks. Based on specific imaging findings, axial T1 post gadolinium and T2 fat saturated or Flair are mostly used to delineate SBC [31].

#### Procedure for CTV(s) delineation

Although the definition of the GTV appears consensual between the participating centers, there are large variations in defining the CTVs and especially the low-risk CTV (Table 2D). Subjective determinants such as clinical experience come into account.

Target volumes may have several names according to the institution; numbering can be misleading in relation with the dose-level applied [29, 30, 32].

#### CTV-PTV margin

In the treatment of cranial lesions, the major source of geometrical deviation is the relative motion of skin and bone anatomy, significantly affecting the repositioning accuracy [33–35]. In addition, the particle range variation is reported to be around 1 mm, representing a further source of systematic error [36].

Most facilities apply a 2–3 mm margin to define the PTV according to two methods: i) isotropic expansion around the related CTV; ii) portal-specific PTV.

### Dose prescription

#### Treatment schedule

The two proposed treatment schedules are reported among the active facilities (Table 2E) [29, 30].

CNAO applies the most hypofractionated dose schedule by default (B); however, whenever the PTV cannot be properly covered, schedule (A) is applied.

#### Dose specification

The European and Japanese teams apply different units and different formulae to specify and calculate equieffective dose (EQD) in relation with their radiobiological modelling.

In Mizoe et al. report, EQD (named “clinical dose”) was expressed in photon-equivalent doses (gray equivalent dose [GyE]). Planning aims to cover the PTV with at least 90% of the prescribed dose [30].

In the HIT-1 protocol, EQD is expressed in GyE. Noteworthy, GSI reported the dose in CGE (cobalt gray equivalent) as well as in Mizoe review [14, 30].

At GSI/HIT/MIT, EQD is rather specified to each scan spot covering the PTV. Throughout the dose distribution, at least 90% of the prescribed dose aims to cover more than 95% of the PTV unless OAR constraints limit the dose. Hot and cold spots never exceed the maximum diameter of 15 mm [10].

In its ULICE-report, CNAO specified dose in Gy [RBE] [32].

#### Biological modeling

At NIRS, the calculation of “clinical dose” is based on the relationships between RBE and LET of human salivary gland tumor (HSG) cells for flattening the SOBP and on the clinical experience with fast neutrons. The RBE of carbon ions is assumed to be 3.0 at the distal region of SOBP [37]. This method was implemented in the initial HiPLAN treatment planning system (TPS) and disseminated to the other Japanese centers [38]. With the implementation of the active scanning delivery method at the NIRS new facility, the modified microdosimetric kinetic model (MKM) was integrated into a novel TPS [39].

In Europe, the RBE is determined for each single spot through the local effect model (LEM) which assumes that equal local energy deposit should lead to equal local effects in the cell nucleus, independently of the radiation quality [40].

#### Dose constraints on OAR

Dose constraints applied to OAR are summarized in Table 2E.

At HIT-MIT, equivalent EQD in 2Gy fractions are retrieved to cope with the corresponding QUANTEC recommendations for optic structures as well as brain stem surface, assuming alpha/beta values of 2Gy.

### Treatment planning

#### Treatment planning system

Overall, there are no major differences on the basic functionalities of the TPS (Table 2F).

The Japanese centers are equipped with XiO-N (ELEKTA, Stockholm, Sweden and Mitsubishi Electric, Tokyo, Japan) with a divergent pencil beam method algorithm based on the modified MKM EQD calculation model since 2012.

In the European centers, the inverse TPS - Syngo RT Planning (Siemens, Erlangen, Germany) is presently used. Syngo RT Planning includes a detailed radiobiological modelling of the effects of high LET radiation based on the LEM model and adopts pencil beam algorithms too.

Noteworthy, CNAO recently equipped with *the Carbon Planning* module on Ray Station (RaySearch Laboratories AB, Sweden) - including modules for carbon pencil beam scanning with robust biological optimization (using both the MKM and LEM models).

### Dose delivery

#### Two delivery systems

The narrow pristine peak produced by the accelerator cannot be used directly so that the beam delivery system is of prime importance. Two strategies have been applied: PS and ABS [41](Table 2G).

Since the beginning of this decade, most CIRT facilities have adopted the latter in order to limit the reduction of the beam fluence and fragmentation. Among the participating centers, HIMAC pilot facility and Gunma still use PS.

#### Beam geometry

In PS, beam parameters such as MLC positions, SOBP size, range shifter thickness, range compensator shape, etc. are adapted to satisfy the tolerance doses for OARs.

With ABS, treatment planning is either possible by single field uniform dose optimization (SFUD) or by multiple field optimization (IMPT, Intensity Modulated Particle Therapy). Two to four irradiation fields are chosen at HIT-MIT.

#### Methods to ensure positioning reproducibility

Positioning accuracy is controlled using orthogonal x-rays or cone-beam-CTs. Set-up deviations are corrected prior to irradiation by correction with the vector of the robotic table. A 6 degrees-of-freedom correction is permitted in the most advanced facilities.

In CNAO optical tracking and in-room imaging systems are integrated [42].

## Discussion and suggestions from ULICE WP2

Van Herk reviewed the most common uncertainties affecting dose distribution in external beam radiotherapy (EBRT) [43]. Particle therapy raises additional issues and uncertainties in dose deposition mainly linked to the finite range as demonstrated in protontherapy [44].

Analyzing the whole CIRT planning process in the participating centers, we pointed out the singularity of the facilities and beam delivery systems, a divergent definition of target volumes, the multiplicity of TPS and EQD calculation approximations. Our multidisciplinary working group proposed the following counter-measures addressing the improvable limitations.

### Beam production and delivery

Differences in equipment between centers may influence the components of the physical dose at the patient’s entrance.

It appears possible to accurately measure the physical dose and its composition in terms of individual particle energy, direction and LET spectrum at the exit of the delivery system. Such parameters in addition to their spatial distribution in relation with cross-section anatomy could be stored to feed more robust modeling systems in the future.

### Immobilization and anatomic data acquisition

devices and the CIRT beam has to be emphasized as reported in our clinical model [45, 46]. Although they cannot easily be standardized, positioning systems and anatomic data acquisition (contrast medium use) must be perfectly described in the trial protocol as this could affect particle range calculation.

### Target volume definition and delineation

The GTV and CTV are oncological volumes defined in the dedicated ICRU Reports [4, 24–28]. Contrarily to GTV, CTV delineation varies substantially between the centers. We could hold a joint symposium to adopt consensus delineation guidelines with standardized imaging procedures as performed in other tumor locations [47] - keeping in mind the natural history of the disease [48, 49].

Conversely to EBRT, adding internal and set-up margins quadratically to construct PTV [4] appears inadequate in CIRT since depth margins are to be incorporated to account for range uncertainties in addition to the typical lateral geometric uncertainties. When using a single beam, the different margins could theoretically combined as an anisotropic shell around the CTV [50]. Portal-specific PTV should be theoretically employed in PS. However, when two (or more) non-parallel opposed particle beams are applied, delineation of a (unique) PTV would generate complex computation issues for the TPS [26]. ABS raise additional issues. The dose is not prescribed to a reference point but to each voxel. Moreover, organ motion, artificial material and tissue heterogeneities may have a strong impact on dose distribution [51, 52]. Overall, specific attention and complex irradiation techniques such as gating, repainting and tracking are needed to alleviate the motion effect in CIRT although this factor is not in the foreground in SBC [53–55].

### Dose specification and calculation

The ICRU recommends that the absorbed dose distribution (3D) be systematically reported, to allow interpretation of the effects and to reconstruct the irradiation conditions if needed [27, 56]. However, specification of these quantities alone is not usually sufficient to predict biological effects when comparing treatments in different conditions. In CIRT the particle energy spectrum varies along the ion path within the treated volume. Each voxel receives a mixed field of particles of different LET and nature due to fragmentation, so the same physical dose at a given point may correspond to different particle spectra, each of which could lead to a particular biological and clinical effect [57].

Despite different specifications, EQD should be reported in addition to absorbed dose [10, 57–59]. These two quantities are expressed in Gy. In order to avoid confusion, the symbol for EQD should be written “D (EQD)” with a space between “D” and “EQD”.

**Fig. 2:**
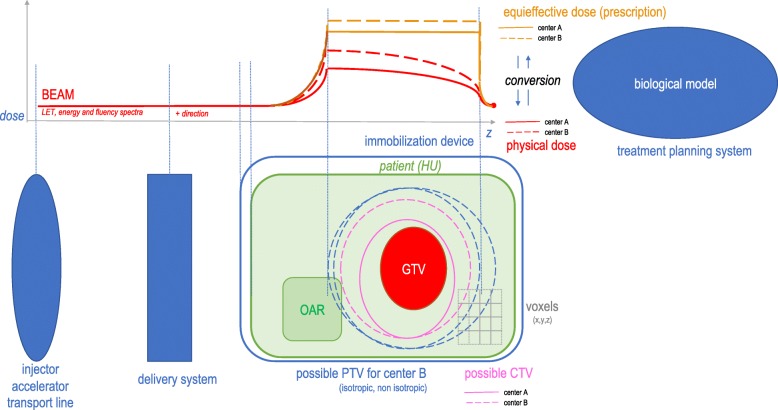
Uncertainties affecting dose deposition throughout CIRT treatment planning process

EQD is the product of absorbed dose and a transformation function (no dimension) which includes all the factors that influence the biological/medical effect(s) – including RBE [19] (Fig. 2). It is likely that the transformation functions are distinct between the centers and their determinants need thus to be reported.

In addition the calculation of EQD is not equivalent between the active facilities and comparing different TPS is a very difficult task in CIRT due to the many interconnected components and the different biological models [60].

**Fig. 3:**
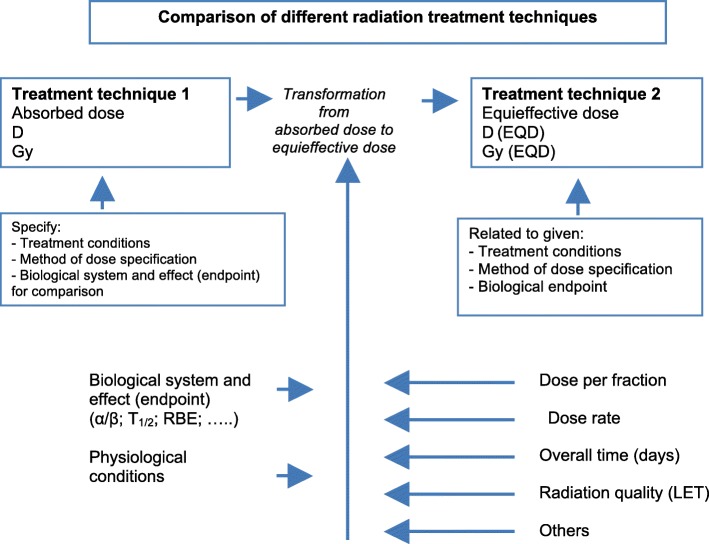
Transformation of absorbed dose D to equieffective dose applicable to all types of radiation therapy techniques. The concept of EQD includes the combined effects of recovery capacity and recovery kinetics

Each individual TPS generate dose calculation uncertainties as soon the beam penetrates into the patient due to the modeling of mixed radiation field and tissue heterogeneities [61] (Fig. 3). Monte Carlo simulations are probably the most accurate and reliable to estimate those uncertainties [62]. NIRS estimated that EQDs produced at NIRS and GSI are biologically identical using HSG and in vivo assays [63]. CNAO thus recalculated their treatment plans - minimizing differences in physical dose distributions between the two treatment plans. FLUKA has been interfaced with LEM I to retrieve physical doses. Overall, the NIRS model estimates a lower RBE of carbon ions when compared to LEM. They proposed conversion tables to minimize the physical dose variations in the PTV [64–66].

Alternative models particularly in the domain of nano- and microdosimetry could apply [67]. A model-independent interface for RBE predictions is being implemented [68].

So far, research in treatment planning of CIRT is usually based on a fixed RBE or α/β value. Since fractionation regimens differ from 3D-CRT, α/β ratios for dose-limiting toxicity and the specific tumor cell type are expected to differ substantially [69]. Radiobiological data for both human tumors and healthy tissues are incomplete. Ideally, prospective experiments should be performed. Overlapping of PTV and OAR raises additional issues [70].

### Quality assurance procedures

Each center has developed its own quality assurance procedures based on local equipment and the available “homemade” measuring tools. When international clinical trials will be initiated, it will be critical to have a uniform quality assurance procedure.

ICRU/IAEA Committee Report 76 - which some of the members of our committee belonged to - proposed recommendations for harmonized dosimetry guidelines and accurate beam calibration [71].

Thereupon, ETOILE is the first transnational prospective randomized trial comparing definitive carbon ion therapy versus photon or combined photon and protontherapy as standard treatment for unresectable or macroscopically uncompleted resected radioresistant tumors. Eligible tumors are axial chordoma (except of base of skull), adenoid cystic carcinoma of head and neck (except of trachea) and sarcomas of any site (except chondrosarcoma of the skull base), non-previously irradiated and without pre-planned surgery or chemotherapy after the clinical trial procedure. Randomization is balanced 1 for 1. Patients of the experimental arm are treated in carbon ions centers in Europe and patients of the standard arm are treated in France in their closest participating radiotherapy center. An accrual of 250 patients is needed and an absolute difference of 20% of relapse free survival at five years is awaited. The main endpoint is the progression free survival at five years (ClinicalTrials.gov Identifier: NCT02838602) We applied the aforementioned proposals when we drafted the protocol [72].

## Conclusion

Multiple uncertainties affect EQD definition, deposition and calculation in CIRT. Although it is not possible to harmonize all the steps of the treatment planning between the active centers, we proposed some of the following suggestions in order to further pool the results in the frame of international trials. Reporting only EQD appears clearly insufficient. Registering and storing raw individual particle number, energy and direction prior entry into the patient and LET in each voxel (for all fragments) according to the simulation CT scan, could allow for centralized EQD reconstruction with existing or novel radiobiological models. On the other hand, it is theoretically possible to standardize the definition and delineation of structures.

## Abbreviations

ABS: Active beam shaping
CIRT: Carbon ion radiotherapy
CNAO: Centro Nazionale di Adroterapia Oncologica
EQD: Equieffective dose
GSI: Gesellschaft für Schwerionenforschung
HIMAC: Heavy Ion Medical Accelerator in Chiba
HIT: Heidelberg Ion Beam Therapy Center
IAEA: International Atomic Energy Agency
ICRU: International Commission on Radiation Units and Measurements
LEM: local effect model
LET: linear energy transfer
MIT: Marburger Ionenstrahl-Therapiezentrum
NIRS: National Institute of Radiological Sciences
OAR: Organ(s) at risk
PS: Passive beam shaping
PTCOG: Particle Therapy Co-Operative Group
RBE: Relative biological effectiveness
SBC: Skull base chordoma(s)
SOBP: Spread-out Bragg peak
ULICE: Union of light ion centers in Europe
